# Infectious phage particles packaging antibiotic resistance genes found in meat products and chicken feces

**DOI:** 10.1038/s41598-019-49898-0

**Published:** 2019-09-16

**Authors:** Clara Gómez-Gómez, Pedro Blanco-Picazo, Maryury Brown-Jaque, Pablo Quirós, Lorena Rodríguez-Rubio, Marta Cerdà-Cuellar, Maite Muniesa

**Affiliations:** 1 Department of Genetics, Microbiology and Statistics, University of Barcelona, Diagonal 643, Annex, Floor 0, E-08028 Barcelona, Spain; 2 IRTA, Centre de Recerca en Sanitat Animal (CReSA, IRTA-UAB), Campus de la Universitat Autonoma de Barcelona, 08193 Bellaterra, Barcelona Spain

**Keywords:** Antimicrobial resistance, Bacteriophages

## Abstract

Bacteriophages can package part of their host’s genetic material, including antibiotic resistance genes (ARGs), contributing to a rapid dissemination of resistances among bacteria. Phage particles containing ARGs were evaluated in meat, pork, beef and chicken minced meat, and ham and mortadella, purchased in local retailer. Ten ARGs (*bla*_TEM_, *bla*_CTX-M-1_, *bla*_CTX-M-9_, *bla*_OXA-48_, *bla*_VIM_, *qnrA, qnrS, mecA, armA* and *sul1*) were analyzed by qPCR in the phage DNA fraction. The genes were quantified, before and after propagation experiments in *Escherichia coli*, to evaluate the ability of ARG-carrying phage particles to infect and propagate in a bacterial host. According to microbiological parameters, all samples were acceptable for consumption. ARGs were detected in most of the samples after particle propagation indicating that at least part of the isolated phage particles were infectious, being *sul1*the most abundant ARG in all the matrices followed by β-lactamase genes. ARGs were also found in the phage DNA fraction of thirty-seven archive chicken cecal samples, confirming chicken fecal microbiota as an important ARG reservoir and the plausible origin of the particles found in meat. Phages are vehicles for gene transmission in meat that should not be underestimated as a risk factor in the global crisis of antibiotic resistance.

## Introduction

Antibiotic resistance is a major threat to public health and food security. A decreasing production of new antimicrobial compounds and a reduced effectiveness of the existing ones caused by the emergence of resistant bacteria is resulting in higher mortality rates^1^. The uncontrolled use of antibiotics and the ability of bacteria to mutate or acquire external genes is leading to the emergence of resistant strains. Epidemiological studies confirm that antibiotic consumption is directly correlated with the emergence and dissemination of resistance^2^.

Natural antibiotics have existed for billions of years in bacterial ecosystems as a strategy for adaptation and defense against other bacteria^3^, resulting in a simultaneous evolution of antibiotic resistance genes (ARGs)^4^. The resistance originated in environmental reservoirs and transmitted to human pathogens^5^ has been exacerbated by exposure to antibiotics present in clinical and agricultural settings^6,7^. Indeed, most of the antibiotics used in medicine, livestock and aquaculture are excreted practically unaltered to the environment, generating a pressure that selects for naturally occurring resistant strains and their accompanying ARG^8^. Bacteria can be intrinsically resistant to antibiotics or can become resistant by mutation or by acquisition of ARGs by horizontal gene transfer^9,10^. While conjugation and transformation are well-studied mechanisms, transduction, which is understood as gene transfer mediated by a bacterial virus or bacteriophage/phage, is attracting increasing attention and has perhaps been underestimated^11,12^.

Three mechanisms of transduction have been described so far; specialized transduction^13^, mediated by temperate phages that lysogenize the bacterial cell and mobilize the genes adjacent to their insertion site. Generalized transduction^14^ mediated by virulent phages that package bacterial DNA instead of phage DNA. And the most recent mechanism described, lateral transduction mediated by temperate phages that do not excise from the bacterial chromosome after induction and generate capsids able to package only bacterial DNA located downstream the phage insertion site with great efficiency^15^. While temperate phages can propagate in a suitable host strain generating phage progeny, transducing particles (containing only bacterial DNA) generated by generalized or lateral transduction, can infect a new host cell but do not possess phage genes and therefore are unable to propagate.

Bacteriophages are globally ubiquitous and extremely abundant, with an estimated 10^31^ bacteriophages in the biosphere^16^. There is also a high percentage (from 4 to 68%) of lysogenic bacteria containing inducible prophages in different ecosystems^17^. Thanks to their structural characteristics, bacteriophages are highly persistent in the environment, the capsid acting as a shield that protects the packaged DNA^18,19^. Phage particles carrying ARGs have been described in a range of environments, including sludge, soil and sewage^20–23^. Their origin in sewage is probably human fecal pollution, including healthy humans and hospital wastes, and animal fecal pollution from farms, which is supported by the detection of ARG-containing phage particles in human and animal feces^24–26^. ARG-containing phage particles have also been found in the human respiratory tract^27^. Thus, the most plausible origin of these particles, whether in the intestines or respiratory tract, is the microbiota^25^. Once free ARG-containing phage particles enter the environment, they can end up in food, as shown in a recent study of vegetables classed suitable for human consumption^28^, and therefore be ingested.

This study focus on the occurrence of ARG-containing phage particles in meat products and, considering that the origin of the particles might be the intestinal microbiota of antibiotic-treated animals, they were also evaluated in chicken cecal samples.

## Results

### Microbiological parameters of the samples

The morphological types of the colonies grown in TSA were quite homogeneous with few differences. Even if we did not identified the different populations of total aerobic bacteria, we assessed that the majority of the aerobic bacteria were resistant to ampicillin (Table 1) with differences close to 0.5 log_10_ units between the number of colonies grown in the absence or presence of ampicillin. Dark blue colonies on Chromocult agar were identified as *E. coli*. These were detected in pork, beef and chicken samples, whereas amp-resistant *E. coli* (dark blue colonies grown on Chromocult agar containing ampicillin) was detected only in pork and chicken (Table 1). In pork and chicken, the differences were of 0.5 and 0.07 log_10_ units, respectively between the number of colonies grown with or without ampicillin, showing also that the largest fraction of the *E. coli* detected was amp-resistant. Somatic coliphages were detected in all samples except mortadella, with an abundance correlating with that of *E. coli*.

**Table 1:** Bacterial and viral indicators.

Microorganism		Pork	Beef	Chicken	Ham	Mortadella
n		10	10	10	5	5
Total aerobic bacteria	%	80	100	100	100	100
	Media	6.2 · 10^5^	4.5 · 10^5^	2.1 · 10^6^	1.0 · 10^5^	2.1 · 10^5^
	SD	3.3 · 10^2^	4.3 · 10^1^	6.7 · 10^3^	1.0 · 10^3^	4.2 · 10^2^
Total aerobic bacteria amp^R^	%	40	100	100	100	100
	Media	2.4 · 10^5^	3.0 · 10^5^	6.3 · 10^5^	6.7 · 10^4^	4.5 · 10^4^
	SD	1.2 · 10^1^	2.0 · 10^1^	7.1 · 10^4^	8.0 · 10^4^	6.6 · 10^4^
*E. coli*	%	30	30	90	0	0
	Media	2.3 · 10^3^	3.7 · 10^1^	3.3 · 10^3^	<41.6	<41.6
	SD	2.7 · 10^3^	—	7.3 · 10^3^	—	—
*E. coli* amp^R^	%	30	0	90	0	0
	Media	6.6 · 10^2^	<41.6	2.8 · 10^3^	<41.6	<41.6
	SD	4.7 · 10^2^	—	6.7 · 10^2^	—	—
Somatic coliphages	%	60	50	100	40	0
	Media	3.2 · 10^2^	6.7 · 10^1^	2.3 · 10^3^	1.9 · 10^1^	<12.5
	SD	2.5 · 10^2^	5.0 · 10^1^	2.1 · 10^3^	8.8 · 10^0^	—

Total aerobic bacteria and *E. coli* grown in the absence or presence of ampicillin were enumerated as bacterial indicators (CFU/25 g) while somatic coliphages were enumerated as viral indicators (PFU/25 g) in the food samples.

SD Standard deviation.

Minced chicken gave the highest percentage of positive samples for all the indicators and the highest average values of all microorganisms (Table 1), followed by minced pork (not all samples were positive for total aerobic bacteria) and minced beef. *E. coli* was not detected in ham and mortadella, whereas somatic coliphages were undetectable in mortadella but present in ham, albeit at very low densities.

### Detection of ARGs in the DNA from phage particles in meat matrices

The ARGs from the DNA extracted from phage particles in each meat matrix were quantified by qPCR. The samples were considered positive when giving amplification values of the target gene within the limit of quantification (LOQ), which was previously defined with standard curves. Those samples outside the LOQ but within the limit of detection and those that did not show any increase in fluorescence in the qPCR cycles (indeterminate samples) were discounted for the study of gene prevalence.

**Figure 1:**
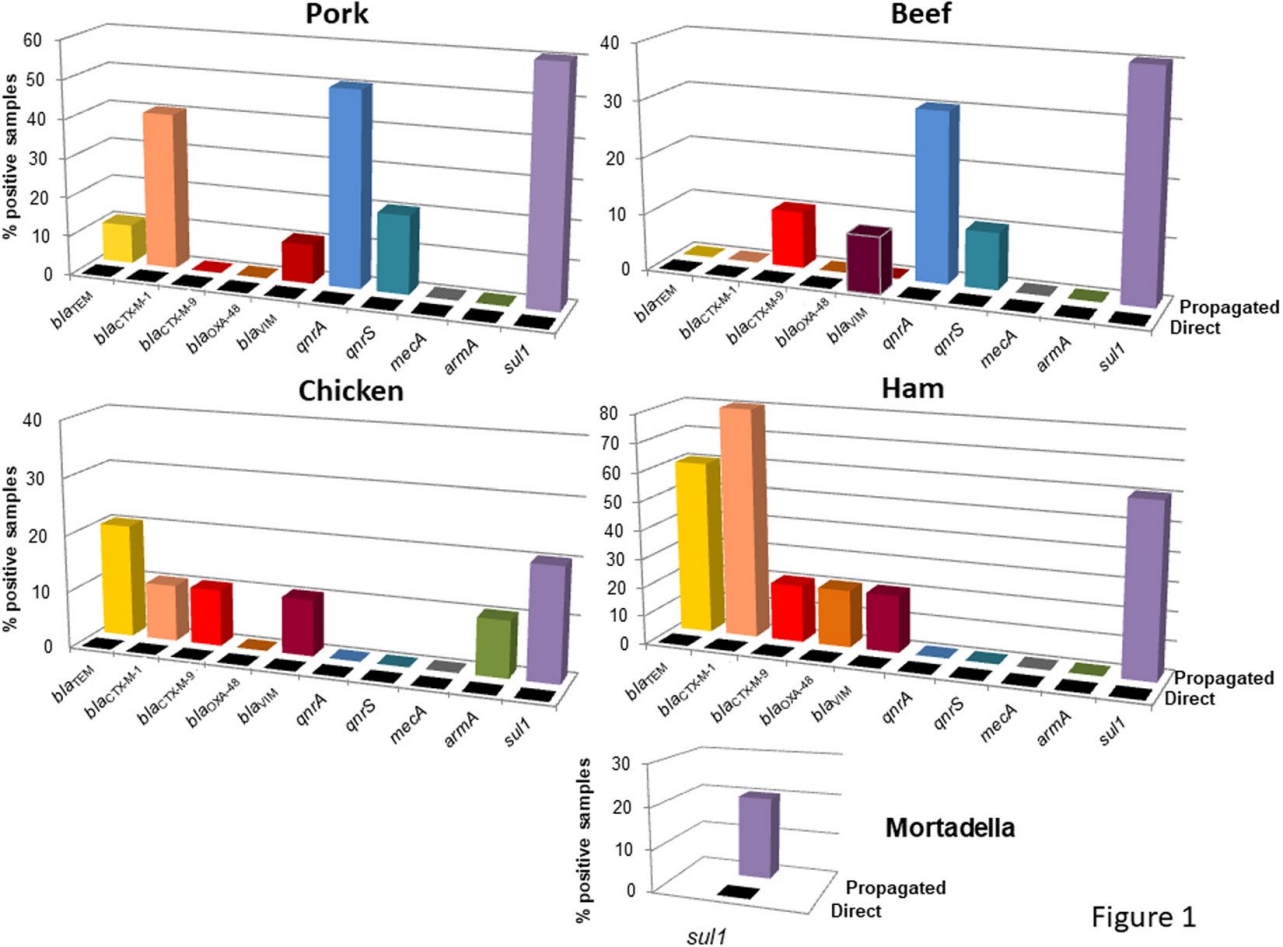
Percentage of positive samples for each ARG in each meat matrix before (direct) and after propagation in the *E. coli* WG5 enrichment culture (propagated). All the values represented are within the limit of quantification.

When ARGs were analyzed in the DNA of phage particles extracted directly from each sample, only *bla*_VIM_ was detected in one beef sample. After propagation, ARGs were identified in DNA from the phage particles in all the matrices, with 10–80% of samples testing positive, depending on the matrix (Fig. 1). The heterogeneous results initially made it difficult to assess which matrix had a higher percentage of positive samples for the different ARGs. In ham, despite only 5 samples being analyzed, 6 ARGs were detected and 4 out of the 5 samples (80%) were positive for *bla*_CTX-M-1_. In contrast, in mortadella only *sul1* was detected, in only one sample (Fig. 1). Among the minced meats, the highest percentages of samples positive for ARGs were in pork (6 ARGs in up to 60% of samples), followed by chicken and beef (6 ARGs in up to 20% and 5 ARGs in up to 40% of samples, respectively). The most prevalent gene was *sul1*, followed by *bla*_CTX-M-1_ and *bla*_TEM_. *bla*_OXA-48_ was only detected in one sample of ham, which was the most polluted meat according to the fecal indicators (presence of total aerobic bacteria: 2.24 × 10^4^ CFU/25 g; absence of *E. coli* but presence of somatic coliphages: 1.25 × 10^1^ PFU/25 g). Accordingly, the same sample of ham also showed the presence of *bla*_TEM,_ *bla*_CTX-M-1_, *bla*_CTX-M-9,_ *bla*_VIM_ and *sul1*.

One chicken sample was positive for *armA*, a prevalent ARG in poultry. This sample also contained fecal pollution indicators (*E. coli* and somatic coliphages), although at below average values (Table 1), as well as *bla*_TEM_ and *bla*_CTX-M-1_. The only tested gene undetected in all the samples of this study was *mecA*.

### Abundance of ARGs in the DNA from phage particles

**Figure 2:**
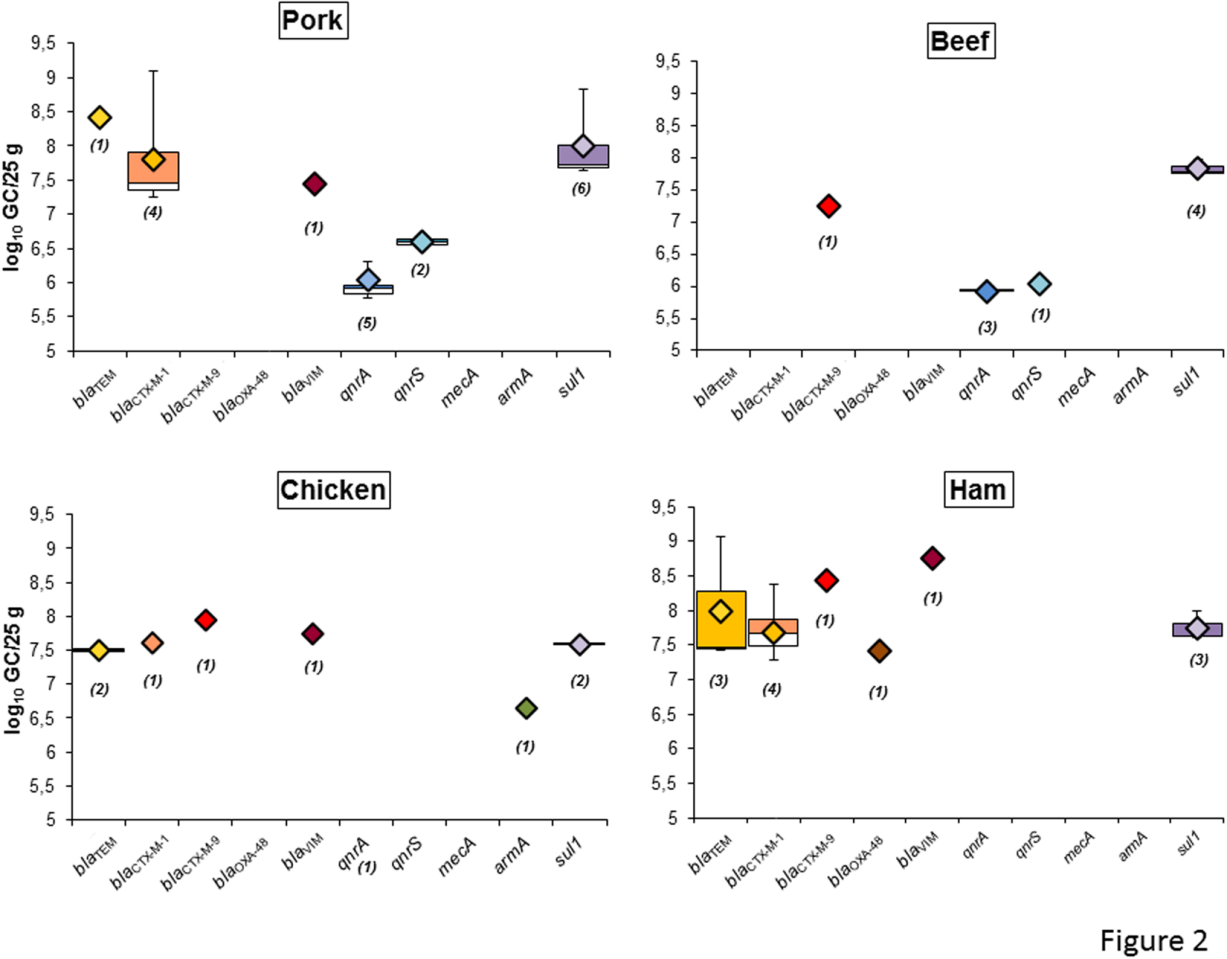
Abundance of ARGs in the DNA isolated from the phage particles of each meat matrix and ham after propagation in the *E. coli* WG5 enrichment cultures. Data is represented in boxplots (log_10_GC/25 g). The diamond represents the average value of the positive samples, the upper squares include samples that present values within the 75th percentile, and in the lower white boxes the values are shown within the 25th percentile. The number of samples appears in brackets.

ARG abundance was evaluated in the samples with levels within the LOQ (Fig. 2). This was determined by the last valid Ct for each ARG assay (Table 2) in the standard curve that is consistent in the diverse replicates.

**Table 2:** Oligonucleotides used in this study for PCR and qPCR assays targeting each ARG. For each assay, the amplimer length and the limit of quantification (LOQ) are indicated.

Target gene	Reaction	Oligonucleotide	Sequence	Amplimer (bp)	LOQ (GC)	Reference
*bla*_TEM_	PCR	UP	CTCACCCAGAAACGCTGGTG	569		^21^
		LP	ATCCGCCTCCATCCAGTCTA			
	qPCR	UP	CACTATTCTCAGAATGACTTGGT	85	7.6	^65^
		LP	TGCATAATTCTCTTACTGTCATG			
		TaqMan TEM	6FAM-CCAGTCACAGAAAAGCATCTTACGG-MGBNFQ			
*bla*_CTX-M-1-_group	PCR	UP	ACGTTAAACACCGCCATTCC	356		^21^
		LP	TCGGTGACGATTTTAGCCGC			
	qPCR	UP	ACCAACGATATCGCGGTGAT	101	8.4	^21^
		LP	ACATCGCGACGGCTTTCT			
		TaqMan CTX-M-1	6FAM–TCGTGCGCCGCTG-MGBNFQ			
*bla*_CTX-M-9-_group	PCR	UP	ACGCTGAATACCGCCATT	352		^26^
		LP	CGATGATTCTCGCCGCTG			
	qPCR	UP	ACCAATGATATTGCGGTGAT	85	13	^26^
		LP	CTGCGTTCTGTTGCGGCT			
		TaqMan CTX-M-9	6FAM – TCGTGCGCCGCTG- MGBNFQ			
*bla*_OXA-48_	PCR	UP	CGTTATGCGTGTATTAGCCTTAT	790		^25^
		LP	TTTTTCCTGTTTGAGCACTTCTTT			
	qPCR	UP	CGGTAGCAAAGGAATGGCAA	133	18.2	^25^
		LP	TGGTTCGCCCGTTTAAGATT			
		TaqMan OXA-48	6FAM-CGTAGTTGTGCTCTGGA-MGBNFQ			
*bla*_VIM_	PCR	UP	TCTACATGACCGCGTCTGTC	748		^28^
		LP	TGTGCTTTGACAACGTTCGC			
	qPCR	UP	AATGGTCTCATTGTCCGTGATG	61	33.9	^28^
		LP	TCGCACCCCACGCTGTA			
		TaqMan VIM	6FAM –TGATGAGTTGCTTTTGATTG- MGBNFQ			
*sul1*	PCR	UP	TTCATGGGCAAAAGCTTGATG	965		^20, 66^
		LP	GGCCGGAAGGTGAATGCTA			
	qPCR	UP	CCGTTGGCCTTCCTGTAAAG	67	5.9	^20^
		LP	TTGCCGATCGCGTGAAGT			
		TaqMan sul1	6FAM-CGAGCCTTGCGGCGG-MGBNFQ			
*mecA*	PCR	UP	GATAGCAGTTATATTTCTA	434		^21^
		LP	ATACTTAGTTCTTTAGCGAT			
	qPCR	UP	CGCAACGTTCAATTTAATTTTGTTAA	92	10.4	^67^
		LP	TGGTCTTTCTGCATTCCTGGA			
		TaqMan mecA	6FAM-AATGACGCTATGATCCCAATCTAACTTCCACA-MGBNFQ			
*qnrA*	PCR	UP	ACGCCAGGATTTGAGTGAC	565		^46^
		LP	CCAGGCACAGATCTTGAC			
	qPCR	UP	AGGATTGCAGTTTCATTGAAAGC	138	8.6	^46^
		LP	TGAACTCTATGCCAAAGCAGTTG			
		TaqMan qnrA	6FAM-TATGCCGATCTGCGCGA-MGBNFQ			
*qnrS*	PCR	UP	AAGTGATCTCACCTTCACCGCTTG	425		^46^
		LP	TTAAGTCTGACTCTTTCAGTGATG			
	qPCR	UP	CGACGTGCTAACTTGCGTGA	118	8.3	^46^
		LP	GGCATTGTTGGAAACTTGCA			
		TaqMan qnrS	6FAM-AGTTCATTGAACAGGGTGA-MGBNFQ			
*armA*	PCR	UP	CAAATGGATAAGAATGATGTT	774		^63^
		LP	TTATTTCTGAAATCCACT			
	qPCR	UP	GAAAGAGTCGCAACATTAAATGACTT	94	33.4	^24^
		LP	GATTGAAGCCACAACCAAAATCT			
		TaqMan armA	6FAM-TCAAACATGTCTCATCTATT-MGBNFQ			
16SrDNA	qPCR	338 F	ACTCCTACGGGAGGCAGCAG	236		^68^
		518 R	ATTACCGCGGCTGCTGG			
pGEM	PCR	pGEM7up	TGTAATACGACTCACTAT			Promega

The abundance of ARGs cannot be considered as an absolute value, because it was ascertained after an enrichment step. Nevertheless, it can be assumed that ARGs with a higher copy number may also have been more abundant in the directly analyzed samples, but at levels below the limit of detection. Also, the detection of ARGs in DNA from phage particles after the enrichment cultures indicates that the phage particles were able to infect and propagate on the host strain and were therefore infectious.

As indicated in the previous section, the only ARG detected in DNA from the phage particles isolated directly from minced beef was *bla*_VIM_, at an abundance of 6.2 log_10_ GC in 25 g of a single sample. This sample showed values of amp-resistant total aerobic bacteria of 2.63 × 10^4^ CFU/25 g, below the average for beef, and an absence of *E. coli* or amp-resistant *E. coli*. The inability of the particles harboring *bla*_VIM_ in this sample to infect *E. coli* WG5 was demonstrated by the absence of the gene after propagation, suggesting that the particles ceased to be infectious or that the strain was an unsuitable host.

As well as being the most prevalent, *sul1* was the most abundant gene on average, reaching densities close to or higher than 10^8^ GC/25 g in all the samples (Fig. 2). In minced pork and ham, *bla*_CTX-M-1_ and *bla*_TEM_ levels were also quite high, reaching 10^9^ GC/25 g (Fig. 2). The genes with the lowest densities were *qnrA* and *qnrS*. The other genes gave very heterogeneous signals. In mortadella, only *sul1* was detected in a single sample, with values of 10^7.7^ GC/25 g after propagation.

### ARGs in DNA in phage particles isolated from chicken feces

We tested 37 archived samples of chicken feces obtained in 2015 from the cecum of broiler chickens at a slaughterhouse covering the area of northeastern Spain. As the chicken meat samples gave the highest values of fecal indicators (Table 1), the ARGs in the phage DNA fraction of chicken fecal samples were evaluated for any correlation with the high levels of ARGs detected in chicken meat.

**Figure 3:**
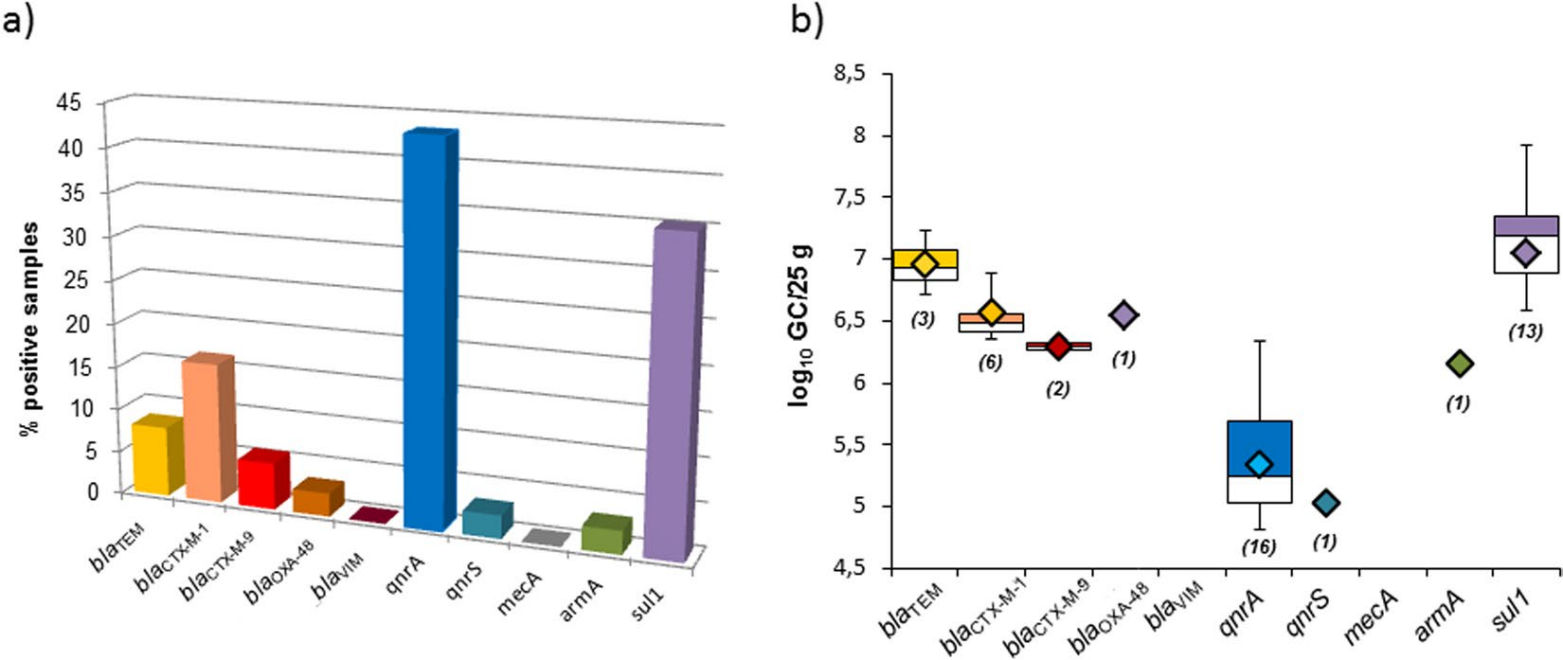
ARGs in phage particles in chicken feces. (**a**) Percentage of positive samples for each ARG in the fecal matrix. All the values represented are within the limit of quantification. (**b**) Abundance of each ARG in chicken feces represented in boxplots (log_10_GC/25 g). The diamond represents the average value of the positive samples, the colored upper squares include samples that present values within the 75th percentile, and in the lower white boxes the values are shown within the 25th percentile. The number of samples appears in brackets.

In this set of samples, microbiological parameters were not analyzed and ARGs were only screened in the DNA of phages directly isolated from the feces without any propagation step. As these archive samples had been frozen for three years, it was considered that viable microorganisms and infectious viruses would have been partially or totally inactivated during storage. In addition, the results (Fig. 3) showed that the concentrations of ARGs in the feces were sufficiently high to be detected without further propagation. The most prevalent genes were *qnrA* and *sul1*, (Fig. 3a), a pattern more similar to the beef and pork samples than to the chicken meat.

The most abundant ARG (Fig. 3b) in chicken feces was again *sul1*. The abundance of quinolone resistance genes was lower, although *qnrA* was present in 43% of the samples. Compared to *sul1* or *qnrA*, the β-lactamases *bla*_TEM_, *bla*_CTX-M-1_ and *bla*_CTX-M-9_ were found in a smaller number of samples, but at high densities of 10^6^–10^7^ GC/25 g. The level of *bla*_OXA-48_ was similar to that of the other β-lactamases in only one sample, and *bla*_VIM_ was not detected at all. As in all the other analyses, *mecA* was not found. Finally, *armA* was detected in only one sample of chicken feces, as occurred in chicken meat, but with a high abundance, only slightly lower than that of the β-lactamase genes (Fig. 3b).

## Discussion

This study focuses on the prevalence of ARGs in bacteriophages, ubiquitous bacterial viruses that have been reported in diverse environments^21,22,29–31^, in human^25^ and animal feces^26^, and more recently in vegetables and agricultural soils^28,32^. Bacteriophages facilitate ARG transmission between bacteria, as transduction does not require direct contact between “donor” and “recipient” cells, although the transfer mechanism is not completely understood. Phages can transmit ARGs through generalized transduction, as reported for *Salmonella* phages in chicken meat^33^ or in veterinary settings^34^. Particles mobilizing ARGs can also be produced in temperate phages induced from bacterial strains^25^, resulting in ARG transfer^35^. Recently a new lateral transduction mechanism has been reported^15^, by which phages package and spread the bacterial chromosome at high frequencies.

Regardless of the mechanism involved, the injection of the mobilized DNA into the recipient strain depends only on the phage capsid. On the other hand, the ability of the transducing particles to propagate in a host cell requires expression of the phage genes, and particles generated through generalized or lateral transduction only possess bacterial DNA. According with previous studies of our group^21,25,28^, transducing particles containing only bacterial DNA (including ARG) are abundant. These results are in accordance with other authors that indicate that mostly bacteriophages, understood as phage capsids containing complete phage genomes, rarely carry antibiotic resistance genes^36^. Instead, transducing particles containing bacterial DNA (including ARG) are not considered when analyzing phage genomes, and might be confounded with bacterial DNA contamination. In the current study however, at least a fraction of the phages were able to propagate, indicating that these were infectious and possessed phage genes in addition to the ARGs. Only one beef sample, in which *bla*_VIM_-containing particles were directly isolated, became negative after the enrichment culture. This case suggests the particles were unable to propagate and the ARG may have been lost after injection in the host cell without a subsequent generation of phage progeny. The infectiveness of the particles is an indication of their ability to attach the recipient cell and inject their DNA, the first steps required for transduction. Our attempts to detect transductants that acquired one of the ARGs were unsuccessful, but this limitation could be explained by several causes: the particles in the samples were able to propagate but not able to transduce the ARG; the recipient host did not possess the proper site to allow recombination of the ARG hence the frequency of transduction was too low to be detected; or even that other virulent phages in the samples propagated and killed the transductant cells before they were isolated, considering that the transducing particles might not generate superinfection immunity in the recipient cell^37^. Probably some of the particles detected in the samples were infectious phages, this is phage capsids with a complete phage genome that incorporated the resistance gene

The food industry has long been subject to a very strict regulation of various microbiological parameters. In minced meat the maximum values of *E. coli* established by the European Union are between 50 and 500 CFU/g^38^. Our samples fulfilled the requirements and did not exceed these values, although their microbiota contained a significant population resistant to β-lactam antibiotics. The detected ARGs exhibited considerable heterogeneity in prevalence and/or abundance. Nevertheless, as indicated above, as the abundance is a consequence of the propagation steps, the densities obtained can only be considered as indicative.

All the samples contained s*ul1* in densities of up to 10^7–8^ GC/25 g. This ubiquity may be explained by the frequent use of sulfonamides in veterinary medicine in the European Union^39,40^. β-lactamase genes were also highly prevalent, again in accordance with current veterinary use^41^ and the pervasiveness of extended-spectrum β-lactamase-producing *Enterobacteriaceae* in animal feces^42–44^ as well as the presence of ARGs in the phage DNA fraction in animal wastewater. Worthy of note is the detection of *bla*_OXA-48_, an emerging carbapenemase that hinders treatment with carbapenems. Besides its clinical significance, it has previously been detected chromosomically in bacteria from food-producing animals^45^ and in DNA in phage particles isolated from agricultural settings^28^. Quinolone-resistance genes, particularly *qnrA*, were prevalent in pork and beef and also present in chicken feces in moderate abundance, in agreement with observations in animal wastewater^46^. Quinolones are frequently administered in cattle and swine^47^, although their use in animals is restricted to the treatment of infections^48^. *armA* was found in chicken meat and feces in moderate levels. It has previously been reported in *Salmonella* or *E. coli* from pigs and poultry, alone or in combination with other ARGs^49^. Finally, the non-detection of *mecA* in our samples could be due to its absence, or to its relation to *Staphylococcus* sp.^50^; phage particles containing *mecA* and generated from *Staphylococcus* might be unable to propagate in *E. coli* and therefore be undetectable.

Both ham and mortadella undergo thermal processing in their preparation, which largely eliminates microorganisms. Further, it is not ruled out that these products contain preservatives that prevent the growth of microorganisms. In spite of similar levels of culturable microbiota, ham showed more infective ARG-phage particles than mortadella. This could be due to an incorrect manipulation in the shop where it was bought, or perhaps because ham is more commonly consumed than mortadella and therefore suffers more manipulation in a single day. Taking into account that each sample was purchased in a different establishment, cross-contamination may be relatively common since at least one ARG was detected in phage particles in all samples.

The Joint Inter-agency Antimicrobial Consumption and Resistance Analysis Spain^47^ estimated the antibiotic consumption by the most relevant animal producing species. The report shows that swine group most treated with β-lactam antibiotics and quinolones (58 and 60% respectively), partially explaining the prevalence and ARG abundance in pork and ham in this study, being cattle the second most treated group (27–28) followed byo poultry (6–7%).

The chicken feces were found to contain ARG-packaging phage particles, as previously observed in human feces^25^, which points towards feces as a source of the phage particles found in chicken meat. Intensive poultry production, one of the fastest growing industries in the world, greatly depends on antimicrobials to prevent and treat diseases. The prophylactic use of antibiotics is difficult to eradicate due to the fear of infections spreading through the chicken farms. However, such an intensive use of antibiotics implies a very high selective pressure and the promotion of resistance transfer events within the chicken microbiota^51^.

The most prevalent genes in the chicken fecal samples were *sul1* and *qnrA*. Sulfonamides and fluoroquinolones are strongly adsorbed to feces^40^, persisting for a long time after excretion and elimination. The constant presence of antibiotics may select these resistances in the fecal microbiota, which are subsequently spread in phage particles. Thus, the fecal contamination in the samples, although low, could be the origin of the ARG-containing phage particles detected. The presence of fecal contamination in chicken meat is attributed to breeding conditions and the processing and handling of the meat before selling. When meat is crushed, microorganisms present on the surface can penetrate the whole product, so thorough cooking is vital before consumption^52^.

Antimicrobials are administered in livestock for preventive and/or therapeutic purposes, but they have also been used as growth promoters to increase the efficiency of animal production, a practice prohibited in Europe since 2006 (EC Regulation No. 1831/2003)^53^. The selective pressure arising from this massive and indiscriminate use of antibiotics enhances the mobility of antibiotic resistance genes (ARGs) and the subsequent appearance of resistant bacteria^54^. Moreover, phages persist to certain treatments used for the processing of food or water samples, such as chlorination, thermal treatment or high hydrostatic pressure (HHP)^55^. Food additives such as EDTA or sodium citrate^46^ may promote the induction of the lytic cycle of certain prophages that will be spread in the food matrix, in the environment or be ingested and reach the human gut, being able to transduce DNA^56^.

At the moment there is no legislation to control antibiotic resistant bacteria or the ARGs and genetic elements that mobilize them. The official incorporation of phage detection methods as indicators is only beginning to be envisaged^57^. Meanwhile, the detection of phages is not yet part of food safety practice, including that of phages potentially capable of transferring ARGs to the natural human microbiota as well as to pathogens.

The expansion of resistances will take the world into a post-antibiotic era, where many common and minor infections will again become life-threatening^1^ unless urgent methods are taken.

Levels of ARGs in the samples were quite heterogeneous. The most striking result was the prevalence and abundance of *sul1* and β-lactamase genes, related with commonly used antibiotics in veterinary medicine, and the non-detection of *mecA*. The propagation experiments showed that part of the phage particles carrying ARGs were infective. Since infection is the first step in ARG transduction, this finding provides further evidence for the role of phage particles in the horizontal transmission of ARGs between bacteria, or between animals through cross-contamination, or to humans via the food chain.

In the absence of any type of food safety regulations aimed at monitoring bacteriophages, their consumption by the population constitutes a health risk factor. Hence, there is a need for further study of ARG transmission by phages through the food chain and the possible transfer of ARGs between commensal bacteria and pathogens.

## Methods

### Samples

The minced meat samples used were classified into 3 groups, pork, beef, and chicken, with 10 samples of each; the sliced meat analyzed consisted of 5 samples of ham and 5 of mortadella. All samples were purchased from local retailers in the area of Barcelona (Spain) during 2017–2018. All of them were considered to be fresh samples as they had not undergone any packaging or freezing process and were analyzed within 24 hours after purchase.

Thirty-seven archived samples of chicken feces (cecal contents) of broiler chickens from different flocks and farms from northeastern Spain were collected at the slaughterhouse in 2015 and stored at −20 °C.

Twenty grams of each sample were homogenized in 60 ml of phage buffer, using the Stomacher homogenizer (IUL Instruments GmbH, Königswinter, Germany) for 2 minutes. Stomacher bags with filters (Afora, Barcelona, Spain) were used to improve the separation of solid waste from the liquid fraction containing the microorganisms.

### Bacterial and viral indicators

Total aerobic microorganisms and total aerobic microorganisms resistant to ampicillin (amp) in the homogenate were evaluated on Tryptone Soy agar (TSA) or TSA with amp (100 μg/ml) at 37 °C.

Total *Escherichia coli* and *E. coli* resistant to amp in the homogenates were determined on Chromocult® Coliform Agar (Merck, Darmstadt, Germany) or Chromocult® Coliform Agar with amp (100 μg/ml) respectively. Incubation was first performed for 2 hours at 37 °C to adapt potentially damaged microorganisms and then overnight at 44 °C. Ten percent of the blue colonies presumed to be *E. coli* were confirmed with the Indole test and grown in McConkey agar.

Somatic coliphages, proposed as viral indicators of fecal pollution^58^, were evaluated to determine the presence of fecal viruses in the samples. Ten ml of homogenates obtained as above were centrifuged for 15 minutes at 4000*xg* and the supernatant was filtered through 0.22 μm low protein binding polyethersulfone membranes (PES) (Millex -GP, Millipore, Bedford, MA). Ten-fold dilutions of the filtrates were analyzed in duplicate for the presence of somatic coliphages following the ISO standard method^59^ that uses *E. coli* strain WG5 (ATCC 700078) as the bacterial host. Plates were incubated at 37 °C for 18 h.

Each homogenate was analyzed for all the indicators in duplicate.

### Chicken feces

Four g of feces were homogenized in 16 ml of phage buffer using a horizontal agitator at 800 rpm for 10 minutes and then centrifuged at 4000* × g* for 25 minutes. The supernatant was collected, and centrifugation was repeated under the same conditions. The final supernatant was filtered through 0.22 μm low protein binding polyethersulfone membranes (PES) (Millex -GP, Millipore, Bedford, MA) and used for the extraction of phage particles and DNA in the particles. In this case, microbial indicators were not analyzed, as these were archival samples kept at −20 °C since their collection in 2015.

### Extraction of phage particles

One ml of the homogenate filtered as described above was treated with chloroform (1:10 (v/v)) to remove possible DNA carrier vesicles. Briefly, chloroform was added to the sample, which was vigorously vortexed for 5 minutes and centrifuged at 16000* × g* for 5 minutes. The supernatant was recovered and incubated with DNase (100 units/mL of the supernatant) to eliminate any free DNA present in the samples outside the phage particles. DNase was inactivated by heating for 5 minutes at 75 °C and an aliquot was taken as a control. These lysates were used for extraction of DNA in the phage particles or to evaluate the infectivity of the phage particles by propagation.

Controls were performed to confirm the removal of DNA by the DNase treatment and the correct inactivation of DNase by heat treatment. The procedure was done as described previously^60^.

### Extraction of DNA in the phage particles

The protocol for DNA extraction was as previously described^61^ with some modifications. After the treatment with chloroform and DNase, aliquots of 50 μl were stored at −20 °C as negative controls for the presence of non-encapsidated DNA. The extraction of DNA from the phage capsids was continued with the remaining phage suspension. To break the capsids and release the genetic material, the samples were treated with proteinase K (20 mg/ml) in 250 μl of proteinase K buffer and incubated for 1 h at 55 °C. Encapsidated DNA was extracted by phenol-chloroform (1:1) (v:v) treatment and the aqueous phase was again treated with chloroform (1:1) (v:v) by centrifuging at the same speed and time as in the previous step. The remaining phenol/chloroform was removed by adding the mixture to Phase Lock Gel Tubes (5- Prime, Hucoa Erlöss, Madrid, Spain) and centrifuging following the manufacturer’s instructions. DNA was precipitated using 100% ethanol and 3M sodium acetate, and resuspended in 100 µl of ultrapure water. DNA was quantified using a Nanodrop ND-1000 spectrophotometer (NanoDrop Technologies, Thermo Fisher Scientifics, Wilmington, DE).

### Amplification of ARGs in DNA of phage particles

Quantitative real-time PCR (qPCR) using TaqMan hydrolysis probes was conducted with the StepOne Real Time PCR System (Applied Biosystems) in a 20 μl reaction mixture with the TaqMan® Environmental Master Mix 2.0 (Applied Biosystems). The results were analyzed with the Applied Biosystems StepOne™ Instrument program.

Ten qPCR assays targeting ARGs were performed in 9 μl of the sample DNA or quantified plasmid DNA. The ARGs were selected from groups relevant in clinics, abundant in the environment, or that confer resistance to different antibiotic groups used in veterinary. These included β-lactamase genesthat confer resistance to β-lactam antibiotics (*bla*_TEM,_ *bla*_CTX-M__-1_ group, *bla*_CTX-M-9_ group, *bla*_OXA-48_ and *bla*_VIM_), two quinolone resistance genes (*qnrA* and *qnrS*), a gene conferring resistance to methicillin (*mecA*), commonly found in *Staphylococcus*^21,46^, *sul1*, which confers resistance to sulfonamides and is frequently found in environmental and clinical bacterial populations^62^, and *armA*, which encodes aminoglycoside resistance and is widely distributed in *Enterobacteriaceae*^63^.

For quantification, standards of known concentration containing a fragment of each ARG were prepared. Each ARG was amplified by conventional PCR, using an Applied Biosystems 2720 Thermal Cycler (Applied Biosystems, Barcelona, Spain) with the primers described in Table 2. The amplicons were purified and cloned into a pGEM-T Easy vector for insertion of PCR products (Promega, Barcelona, Spain). The correct cloning of the fragment in the vector was confirmed by PCR using primer pGEM7up (Table 2) and the construct was used to generate the standard curves as previously described^21^. The standards were also used as positive controls.

All samples were performed in triplicate (including the standards and negative controls). The number of gene copies (GC) was defined as the mean of the triplicate data obtained. To quantify the ARGs, we considered the GC results obtained within the threshold cycle (Ct) within the limit of quantification (LOQ).

### Negative controls to exclude contamination with non-encapsidated DNA

To rule out contamination with DNA outside the phage particles (either bacterial or free DNA present in the samples), the aliquot of each sample taken after DNase treatment and before desencapsidation by proteinase K digestion, and kept at −20 °C, was screened by qPCR for each ARG and for bacterial 16SrDNA (Table 2). These controls were expected to be negative after the removal of all non-encapsidated DNA by the DNase. The ARGs were evaluated by qPCR assays as described above and in Table 2, and the absence of bacterial 16S rDNA was verified by qPCR using Power SYBR Green PCR Master Mix (Thermo Fisher Scientific) and primers 338F/518R (Table 2).

### Propagation cultures

Phage particles in the samples were propagated in enrichment cultures of the strain *E. coli* WG5 as a host bacterium. This strain, used for the somatic coliphage count, was selected because of its sensitivity to phage infection^28^ and because it does not harbor any of the ARGs targeted in this study or any prophage^64^.

To evaluate the infectivity of the phage particles carrying ARGs, the analysis of ARGs by qPCR was carried out both before (direct quantification from the sample) and after phage propagation in the enrichment cultures. As the chicken feces were archive samples and not expected to contain infectious phages, the propagation experiments were only conducted with phages in the meat samples. If phages carrying ARGs were able to infect and propagate on the host strain, the ARG levels would be expected to increase due to the higher concentration of phage particles. If ARG levels decreased, the particles would be considered incapable of propagating.

The propagation culture was prepared with one ml of phage particles after filtration and DNAse treatment and one ml of *E. coli* WG5 at the exponential phase (OD_600_ 0.3) in 8 ml of Luria-Bertrani broth (LB). This mixture was incubated for 18 h at 37 °C with shaking. After incubation, the phages were purified, and DNA in phage particles was extracted as indicated above.
